# Schneiderian Membrane Elevation up to 8 mm by Transcrestal Technique Using Hollowed Osteotomes: A Human Cadaver Study

**DOI:** 10.1111/cid.70096

**Published:** 2025-10-11

**Authors:** Jean‐Christophe Coutant, Adrien Naveau, Yves Lauverjat, Bruno Ella

**Affiliations:** ^1^ Pôle de Médecine et Chirurgie Bucco‐Dentaire CHU de Bordeaux Bordeaux France; ^2^ UFR des Sciences Odontologiques, Département des Sciences Anatomiques Université de Bordeaux Bordeaux France; ^3^ Laboratoire d'Anatomie Université de Bordeaux Bordeaux France; ^4^ INSERM, BioTis, U1026 Univ. Bordeaux Bordeaux France; ^5^ UFR des Sciences Odontologiques, Département de Prothèses Université de Bordeaux Bordeaux France; ^6^ UFR des Sciences Odontologiques, Département de Parodontologie Université de Bordeaux Bordeaux France

**Keywords:** Schneiderian membrane detachment, sinus floor augmentation, sinus osteotomes, transcrestal technique elevation

## Abstract

**Purpose:**

The objective of this study was to compare the detachment surfaces of Schneiderian membrane elevation of 4–8 mm by transcrestal technique using hollowed osteotomes on human cadavers with 4 mm residual bone height.

**Methods:**

The study followed the CACTUS (ChAracteristics of Cadaver Training and sUrgical Studies) guidelines. Thirty‐four heads with maxillary bilateral edentulous posterior zones and type IV bone (a residual bone height of 4 mm) were selected using cone beam computed tomography (CBCT) scans. Heads were randomly allocated to a group and both sinuses received the same treatment. A sinus floor elevation was performed on the 68 sites using new hollowed osteotome (C.M.C Tech, IBS Implant, Daejeon, South Korea). Samples were divided into two groups: Group 1: sinus elevation of 4 mm; and Group 2: sinus elevation of 8 mm. A postoperative CBCT was performed to analyze membrane integrity and to assess the membrane detachment in mesiodistal and buccopalatal plane.

**Results:**

Of the 68 membrane elevations, only 4 were associated with perforations; 2 occurred in each group. The detachment surface variations between group 1 and group 2 were linear in all directions (Pearson correlation coefficient *ρ* = 0.99). The Student's *t*‐test unveiled significant differences in detachment surfaces between group 1 and group 2 in all directions (*p* < 0.001). The ratio of detachment surface group 2/group 1 was homogeneous in all directions (1.5 ≤ *r* ≤ 1.54). The quality of Schneiderian membrane elevations of 4–8 mm by transcrestal technique was similar using the hollowed osteotomes on human cadavers with 4 mm residual bone height.

**Conclusions:**

In a transcrestal approach using this new‐generation osteotome, sinus membrane elevations of 8 mm were performed with a low perforation rate in cadavers. The use of the new hollowed osteotome allowed a Schneiderian membrane elevation of up to 8 mm in a safe and reproducible manner. This transcrestal elevation protocol could increase the indications of the technique, especially concerning single implant restorations and for frail patients with significant medical history for whom a lateral approach would be too invasive.

## 1 Introduction

Implant restorations in the posterior maxillary regions frequently necessitate addressing the challenge of limited bone height beneath the sinus cavities [1, 2]. In these circumstances, short implants (≤ 6 mm) represent a viable treatment, but they exhibit greater variability and diminished predictability in survival rates than augmentation procedures [3]. Indeed, when less than 8 mm of bone is available below the maxillary sinus, sinus floor elevation techniques should be considered [4]. Maxillary sinus augmentation involves the elevation of a surgical flap, followed by accessing the sinus cavity through a created window. This procedure includes the elevation of the Schneiderian membrane above the maxillary floor and beneath the membrane itself, with the aim of augmenting the bone height and establishing a confined space for subsequent implant placement [5]. The lateral approach (“sinus lift”) is effective and safe [6], but is associated with a postsurgical period that requires more monitoring, high cost, and complex procedures [7, 8, 9]. Nevertheless, the transcrestal approach (“Summers' technique”) offers distinct advantages, including reduced trauma, minimal incision size, decreased operative duration, a short period of healing of the surgical site, and enhanced patient satisfaction [10, 11].

The transcrestal approach consists of pushing the intra‐alveolar bone upwards and thereby fracturing the floor of the sinus to increase the bone height [12, 13]. This procedure can be performed with the addition of grafting materials inserted immediately before the implant [14, 15].

However, perforation of the Schneiderian membrane is the most commonly reported surgical complication, often resulting from procedures performed at the threshold of mechanical stress, thereby leading to membrane breach [16, 17]. This occurs when local tension surpasses its capacity for extension, a phenomenon closely linked to the condition of the membrane and anatomical variations in the morphology and contours of this confined space [9, 18]. An average tension of 7.3 N/mm^3^, generated by a force exerted and concentrated on a point of the fractured osseous block, causes perforation of the sinus membrane [19]. As current transcrestal osteotomes feature a solid rod edge at their tip in a cup design, the forces are concentrated at the center of the cup, creating significant local tension that can overstretch the sinus membrane [4, 20]. Thus, the indication of the technique has been limited to membrane elevation of 4 mm and to residual bone height of 7–9 mm [4, 20, 21].

**FIGURE 1:**
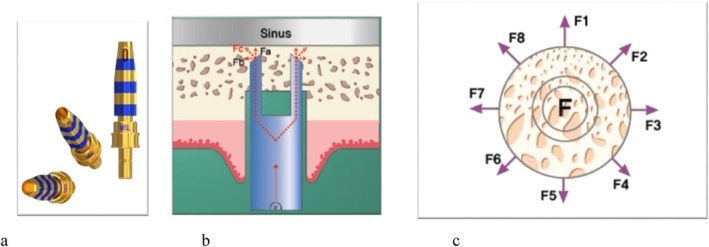
Hollowed osteotome (Magic Sinus Lifter, IBS Implant). (a) Commercial illustration of the hollowed osteotome. (b) Commercial explanation of the osteotome functioning (the osteotome tip is placed 2 mm beneath the sinus membrane, and the bone disc is elevated by the operator's applied pressure). (c) Force dispersion around the osteotome at the bone level (at the level of the osteotome's circumferential cutting edge, the bone is pushed outward). Illustrations from IBS Implant.

Recently, a new generation of osteotomes has become available, with a tip designed as a hollow head (crestal approach with membrane control osteotome, Magic Sinus Lifter, IBS Implant, Daejeon, South Korea; Figure 1). The round front blade with an empty space of 3 mm cuts the bone block in a round shape larger than the diameter of the circular blade [22]. The cut bone block remains attached to the membrane, and when lifted, the osteotome forces are not concentrated at any point but spread in a radial direction from the circular bone fragment. The membrane is elevated and detaches from the osseous floor with a lower tensile/tear strength than with classic Summers osteotomes, which have a dense tip.

The hypothesis of this study was that using these new hollowed osteotomes could allow a Schneiderian membrane elevation of up to 8 mm, in a safe and reproducible manner. The main objective was to compare the detachment surfaces of Schneiderian membrane elevation of 4–8 mm by transcrestal technique using hollowed osteotomes on human cadavers with 4 mm residual bone height. The secondary objective was to compare the amount of membrane detachment in all directions around the implant site after both procedures.

## 2 Materials and Methods

The study adhered to the CACTUS (Characteristics of Cadaver Training and Surgical Studies) guidelines [23]. The principal components of these guidelines are detailed in Table S1. The research was conducted on edentulous maxillae sourced from fresh human cadavers at the Anatomy Laboratory of the University of Bordeaux. The samples were obtained through body donations to the Anatomy Laboratory following the approval of the study protocol in accordance with the University of Bordeaux. The bodies were frozen at −20°C immediately after death and subsequently thawed at room temperature for 24–36 h, depending on the season. Each anatomical specimen was assigned a unique identification number.

Following thawing, the oral cavities were thoroughly rinsed with warm water before the specimens underwent an initial cone beam computed tomography (CBCT) scan to assess residual bone height. CBCT were performed on available heads samples (NewTom VGi evo, NewTom Cefla S.C., Imola, Italy) and analyzed with Osirix software (Pixmeo, Bernex, Switzerland). Thirty‐four heads with bilateral posterior edentulous maxillae and a residual bone height of 4 mm were selected. The type IV bone density was assessed visually by CBCT examination and categorized according to the Lekholm and Zarb classification [24, 25]. Among the available specimens, only those with a residual bone height of 4 mm were included; however, a tolerance of ±0.2 mm was accepted to account for minor measurement variability. Samples displaying anatomical variations, such as intraosseous lesion of the sinus, septa, or dental residues in the sub‐sinus area, were excluded.

In the axial plane, the inter‐incisal foramen served as the reference point, alongside the anterior and posterior landmarks of the sinus, for conducting measurements. The distance between these points was determined utilizing Osirix (Pixmeo, Bernex, Switzerland) software. In the coronal plane, the apex of the bone crest and the sinus floor were employed to calculate the bone crest height, whereas the palatal and buccal walls were utilized to assess the width. The computed measurements that facilitated the determination of the surgical area were transposed onto the bone ridges using a digital caliper (Absolute Series 500, Mitutoyo, Sakado, Japan).

Two experimental groups were compared:

- Group 1: sinus elevation of 4 mm (*n* = 34).
- Group 2: sinus elevation of 8 mm (*n* = 34).

The allocation of each head to a group was done randomly after inclusion by a laboratory technician who did not have access to the radiological data. However, both sinuses from the same head were assigned to the same experimental group.

### 2.1 Surgical Procedure

Surgical procedures were performed 1–2 days later (after which the specimens were returned for a postoperative CBCT to evaluate membrane elevation and integrity). As thawed specimens begin to degrade after approximately 72 h, all procedures were completed within this time frame to ensure optimal working conditions.

All procedures were executed by a single experienced surgeon. A full‐thickness flap was elevated along the entire edentulous ridge on both the left and right sides. A transcrestal sinus floor elevation was performed employing the C.M.C Tech method (C.M.C Tech, IBS Implant). In summary, a drill (Magic Marking Drill, IBS Implant) was utilized to demarcate the site designated for implantation. This initial drilling was conducted to a depth of less than 2 mm, followed by a second drilling up to 2 mm below the sinus floor. The drilling speed was set at 1500 rpm, in accordance with the manufacturer's recommendations. A manual 4.5 mm‐diameter sinus‐osteotome (Magic Sinus Lifter, IBS Implant, Figure 1) enabled to push the sinus floor axially. The osteotomes were slightly cylindrical–conical (Figure 1a), with a proximal diameter of 4.5 mm and a narrower apical diameter of 3.5 mm, designed to facilitate primary stability of the cylindrical implants. The osteotome was used with fins oriented in the mesiodistal direction. In each sinus, the created volume was filled with large particles of demineralized bovine bone mineral (1–2 mm diameter, DBBM Bio‐Oss, Geistlich Pharma AG, Schlieren, Switzerland). The amount of graft material, with a maximum of 0.3 g, was approximately proportional to the height of sinus membrane elevation [26], as grafting was discontinued once the surgeon judged the filling to be clinically optimal. Then, 7 mm‐long implants were placed in Group 1 and 11 mm‐long implants in Group 2 (MagiCore, IBS Implant, both with a 4.5 mm diameter). They were placed using a reducing contra‐angle and a motor (2520RPB and Implanteo 11200, Anthogyr, Sallanches, France) at 15 rpm with irrigation. The implants were used to determine the vertical axis of the reference frame in which the detachment values were calculated.

A postoperative CBCT was performed as previously to analyze membrane integrity and to assess the membrane detachment, as described below. CBCT reconstructions were initially performed in the axial plane, followed by reconstructions in both the mesiodistal plane and the buccopalatal or coronal plane.

### 2.2 Assessment of Membrane Detachment

**FIGURE 2:**
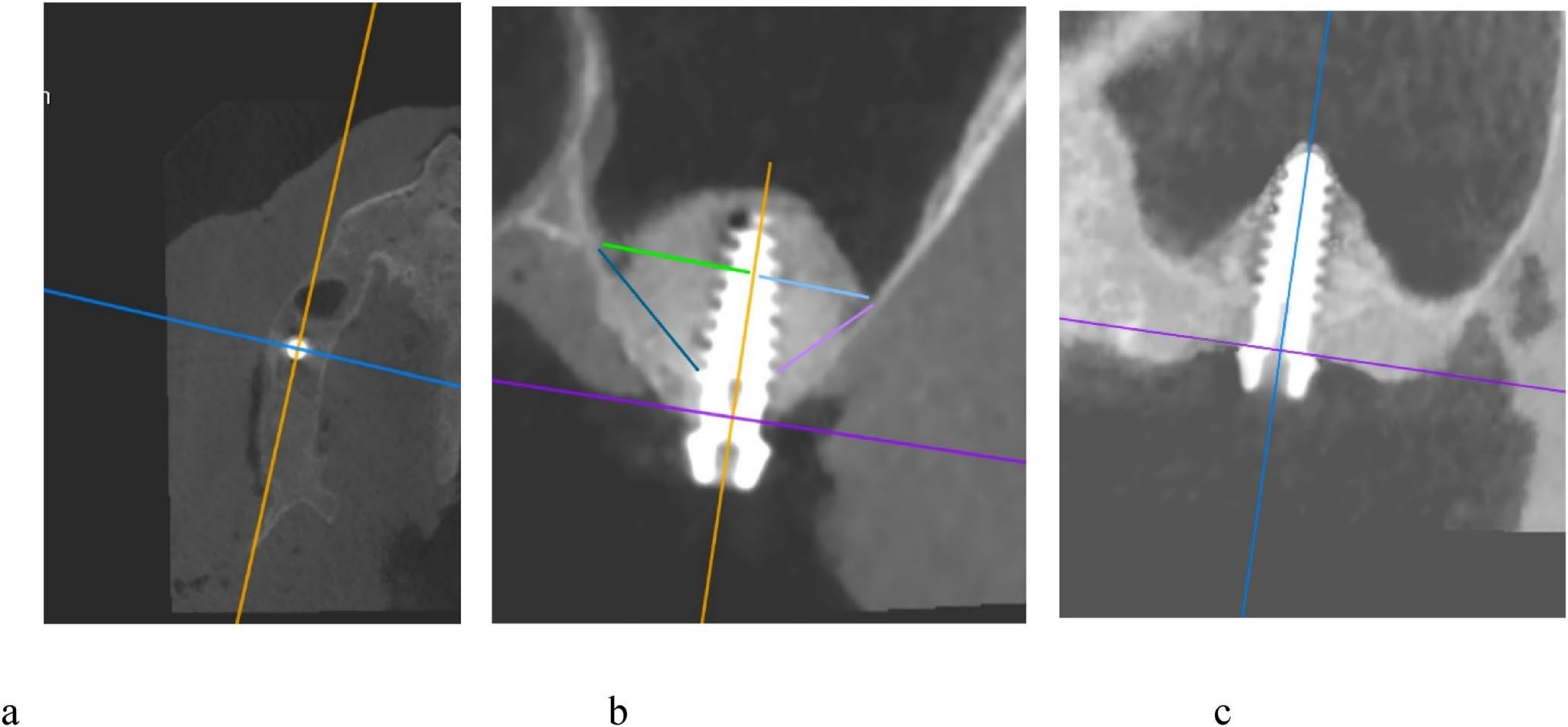
Assessment of membrane detachment. A reference framework was established on the postoperative CBCT to ensure standardization at each site. (a) Mesiodistal (orange line) and buccopalatal (blue) axes were defined in the axial plane. (b) Vertical (orange) and mesiodistal (purple) axes, buccal (light blue) and palatal (green) orthogonal projections were defined in the coronal plane, and detachment occurred on buccal (light purple) and palatal (dark blue) surfaces. (c) Mesial–distal detachments were measured in the mesiodistal axis (vertical axis in blue and mesiodistal axis in purple).

A reference framework was established on the postoperative CBCT to ensure standardization at each site. This was defined as follows:

– From the axial reconstruction plane, a guideline was traced along the midpoint of the alveolar crest and through the center of the implant in a mesiodistal direction, establishing the mesiodistal axis, which intersects with the vertical axis (implant axis). These two axes delineated the mesiodistal plane (Figure 2a).
– An additional line was drawn passing through the center of the implant, orthogonal to both the mesiodistal and vertical axes, thereby establishing the buccopalatal axis. These two axes defined the buccopalatal plane (Figure 2a).

Two detachment points (buccal and palatal) were evaluated along the buccopalatal axis in the coronal plane. Subsequently, the distances between these buccal/palatal detachment points and their orthogonal projections onto the vertical axis were calculated (Figure 2b). Two other detachment points (mesial and distal) were assessed on the mesiodistal axis, and then the measure of the distance between the mesial/distal detachment points and their orthogonal projections on the vertical axis was computed (Figure 2c). All data were compiled into an Excel spreadsheet.

### 2.3 Statistical Analyses

Statistical analyses were conducted utilizing Prism software (GraphPad Software Inc., San Diego, California, USA). Considering that observations within the same cadaver were not independent, Student's *t*‐test for paired variables were used with 62 degrees of freedom. The values for both groups adhered to a normal distribution, thereby satisfying all the prerequisites for parametric testing. Consequently, a Student's *t*‐test was employed to compare two independent values for the same parameter (dimension value determined on each reference frame axis), which included: the M (mesial detachment value), D (distal detachment value), MD (M + D; mesiodistal detachment value), B (buccal detachment value), L (palatal detachment value), and BP (B + P; buccopalatal detachment value) in Groups 1 and 2.

## 3 Results

**FIGURE 3:**
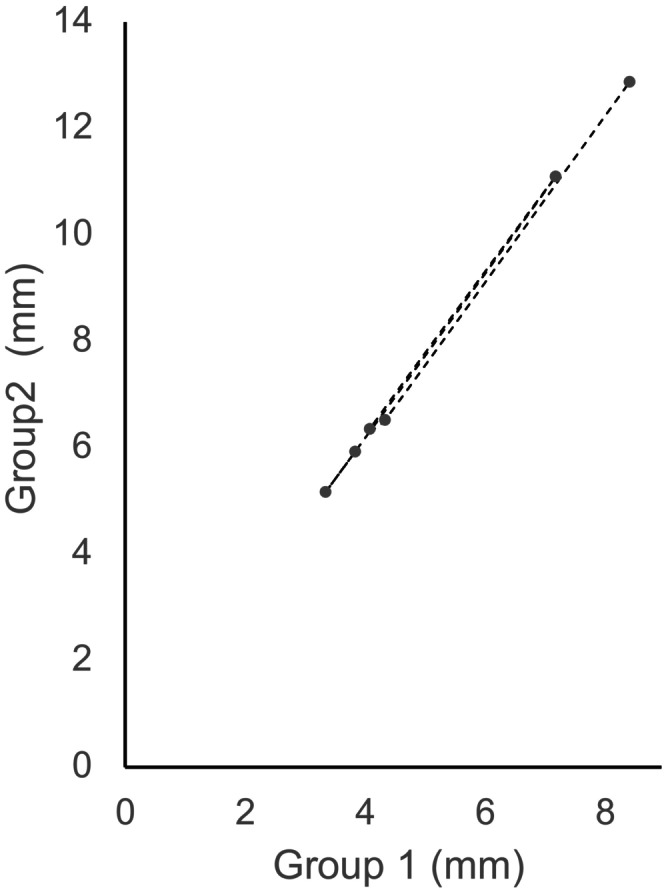
Correlation of the detachment values variations occurring between a 4 mm and an 8 mm elevation. The detachment surface variations between Groups 1 and 2 were linear in all directions, as shown by the Pearson correlation coefficient; *ρ*: 0.9997; 95% confidence interval [0.9968–1].

All specimens included in the study were classified as having type IV bone density based on the CBCT evaluation, according to the Lekholm and Zarb classification [24, 25]. Of the 68 membrane elevations, only 4 were associated with perforations (Tables 1 and 2). Two occurred in Group 1 (Table 1), and two in Group 2 (Table 2). These four failed cases were discarded for the membrane detachment evaluation, resulting in a total of 32 sites in each group. The detachment means in Group 1 (4 mm elevations) were of 3.86 ± 0.98 mm in the buccal direction, 3.37 ± 0.90 mm in the lingual direction, 4.11 ± 1.13 mm in the mesial direction, and 4.36 ± 1.29 mm in the distal direction (Table 1). The detachment means in Group 2 (8 mm elevations) were of 5.93 ± 1.36 mm in the buccal direction, 5.17 ± 1.20 mm in the lingual direction, 6.36 ± 1.05 mm in the mesial direction, and 6.52 ± 1.58 mm in the distal direction (Table 2). The detachment surface variations between Groups 1 and 2 were linear in all directions, as shown by the Pearson correlation coefficient (Figure 3; *ρ* = 0.99). The statistic Student test unveiled significant differences in detachment surfaces between Groups 1 and 2 in all directions (*ρ* < 0.001, Table 3). The ratio of detachment surface Group 2/Group 1 was homogeneously around 1.5 in all directions (1.5 ≤ *r* ≤ 1.54, Table 4).

**TABLE 1:** Values of membrane detachment surfaces assessed on CBCT in all directions for Group 1 (4 mm elevations, in mm).

Subject no.	Site	B	L	BL	M	D	MD
1	27	3.80	2.75	6.55	2.50	3.10	5.60
	14	3.00	2.12	5.12	3.00	5.00	8.00
3	15	2.90	3.00	5.90	2.80	2.30	5.10
	25	4.00	3.00	7.00	2.50	2.30	4.80
4	16	5.70	4.00	9.70	3.30	4.00	7.30
	26	2.70	4.00	6.70	4.00	4.00	8.00
6	17	2.00	1.80	3.80	2.20	2.00	4.20
	26	4.90	2.80	7.70	3.40	3.40	6.80
8	16	3.70	3.20	6.90	4.10	4.00	8.10
	24	2.90	2.60	5.50	4.50	4.30	8.80
10	15	4.40	3.50	7.90	3.40	3.50	6.90
	26	4.50	3.00	7.50	4.00	3.60	7.60
13	17	3.20	3.30	6.50	4.00	5.00	9.00
	27	3.50	2.50	6.00	2.80	3.00	5.80
15	14	5.00	4.40	9.40	6.00	6.00	12.00
	24	4.60	4.70	9.30	5.20	5.70	10.90
16	15	4.80	3.60	8.40	5.00	5.10	10.10
	24	3.50	5.00	8.50	5.00	5.10	10.10
19	14	2.50	2.90	5.40	3.40	3.30	6.70
	25	4.60	3.30	7.90	3.80	3.30	7.10
21	16	4.70	3.60	8.30	4.10	5.00	9.10
	27	5.20	6.00	11.20	4.20	4.80	9.00
23	17	2.80	3.30	6.10	3.10	2.80	5.90
	26	2.90	2.70	5.60	2.50	2.50	5.00
25	16	3.80	4.00	7.80	6.00	5.00	11.00
	26	3.70	3.00	6.70	5.00	5.20	10.20
27	15	Perforation					
	25	5.00	2.90	7.90	5.80	6.00	11.80
29	14	4.00	3.80	7.80	3.50	5.80	9.30
	25	Perforation					
31	15	4.80	2.70	7.50	4.20	6.00	10.20
	26	4.60	2.20	6.80	6.00	6.50	12.50
33	17	2.20	3.00	5.20	6.00	6.00	12.00
	26	3.60	4.40	8.00	4.50	4.70	9.20
*n* = 17	*n* = 34	*n* = 32	*n* = 32	*n* = 32	*n* = 32	*n* = 32	*n* = 32
Mean		3.86	3.37	7.23	4.11	4.36	8.47
Standard deviation		0.98	0.90	1.56	1.13	1.29	2.31
Confidence intervals		[3.38; 4.34]	[2.92; 3.81]	[6.45; 8]	[3.55; 4.67]	[3.72; 5.00]	[7.32; 9.61]

*Note:* Two perforations occurred and were discarded, resulting in 32 sites.

**TABLE 2:** Values of membrane detachment surfaces assessed on CBCT in all directions for Group 2 (8 mm elevations, in mm).

Subject no.	Site	B	L	BL	M	D	MD
2	14	Perforation					
	27	8.00	4.70	12.70	5.00	6.80	11.80
5	25	5.30	4.00	9.30	5.00	5.00	10.00
	15	4.50	4.50	9.00	5.00	5.40	10.40
7	15	6.30	4.00	10.30	7.50	7.70	15.20
	26	6.20	3.60	9.80	7.00	7.10	14.10
9	25	4.00	4.65	8.65	6.00	6.20	12.20
	16	3.10	4.20	7.30	6.20	5.60	11.80
11	14	5.70	4.80	10.50	6.50	6.50	13.00
	24	6.00	5.00	11.00	5.60	5.60	11.20
12	15	6.80	4.70	11.50	6.40	7.80	14.20
	26	4.40	6.00	10.40	6.70	6.00	12.70
14	15	6.90	5.20	12.10	8.20	12.00	20.20
	25	7.50	5.00	12.50	8.20	8.50	16.70
17	15	5.87	5.50	11.37	6.24	8.27	14.51
	27	6.28	4.22	10.50	6.90	6.60	13.50
18	24	5.20	6.40	11.60	5.70	5.70	11.40
	15	9.00	4.80	13.80	6.30	8.00	14.30
20	26	5.28	5.40	10.68	7.00	5.40	12.40
	16	6.20	2.90	9.10	4.90	5.80	10.70
22	17	5.60	3.30	8.90	7.20	7.20	14.40
	25	Perforation					
24	17	5.30	6.30	11.60	4.00	5.80	9.80
	24	8.70	4.80	13.50	7.50	7.50	15.00
26	14	5.00	8.10	13.10	6.00	5.00	11.00
	16	7.50	6.40	13.90	6.00	8.00	14.00
28	17	6.80	5.10	11.90	7.40	7.30	14.70
	27	6.60	5.30	11.90	7.50	5.60	13.10
30	25	4.60	7.00	11.60	4.80	4.20	9.00
	27	6.10	8.10	14.20	6.20	7.30	13.50
32	14	3.40	6.20	9.60	5.50	3.80	9.30
	26	6.00	5.00	11.00	7.00	6.50	13.50
34	15	5.30	4.70	10.00	7.73	4.30	12.03
	26	6.30	5.60	11.90	6.20	6.30	12.50
*n* = 17	*n* = 34	*n* = 32	*n* = 32	*n* = 32	*n* = 32	*n* = 32	*n* = 32
Mean		5.93	5.17	11.10	6.36	6.52	12.88
Standard deviation		1.36	1.20	1.68	1.05	1.58	2.29
Confidence intervals (95%)		[4.57; 7.29]	[3.97; 6.37]	[9.42; 12.78]	[5.31; 7.40]	[4.95; 8.10]	[10.59; 15.17]

*Note:* Two perforations occurred and were discarded, resulting in 32 sites.

**TABLE 3:** Null hypothesis testing.

Student test	B	L	BL	M	D	MD
Ho	Rejected	Rejected	Rejected	Rejected	Rejected	Rejected
*p*	2.51E−08	6.90E−08	1.51E−11	1.66E−09	9.84E−7	8.21E−09

*Note:* The statistic Student test unveiled significant differences in detachment surfaces between Groups 1 and 2 in all directions, rejecting the null hypothesis.

**TABLE 4:** Assessment of the detachment gain for Group 2 versus Group 1 in all directions.

	B	L	BL	M	D	MD
Group 1 (mean value, mm)	3.86	3.37	7.23	4.11	4.36	8.47
Group 2 (mean value, mm)	5.93	5.17	11.10	6.36	6.52	12.88
Ratio	**1.54**	**1.54**	**1.54**	**1.55**	**1.50**	**1.52**

## 4 Discussion

The hypothesis of this study was validated, as use of the new hollowed osteotome allowed a Schneiderian membrane elevation up to 8 mm, in a safe and reproducible manner. The quality of Schneiderian membrane elevations of 4 and 8 mm by transcrestal technique was similar using the hollowed osteotomes on human cadavers with 4 mm residual bone height.

The transcrestal approach indications are currently limited by the risks of membrane perforations, as the result of the tensions exerted by the osteotome on a membrane with limited stretching properties. The membrane perforation rates published in the literature showed controversial data and vary from 2% to 4% [27] to 28% [28], but most long‐term longitudinal studies reported a success rate around 95% using the transcrestal technique with a residual bone higher than 5 mm [29, 30]. In the present study, the global perforation rate was 6%, which is consistent with the literature [27]. In Group 2 with 8 mm elevation, only two perforations occurred, which is below the literature reports about membrane elevations [11, 18]. As the protocol for elevation was conventional, the hollowed osteotome might explain these interesting results.

During routine clinical practice, perforations are assessed by clinical test (the Valsalva maneuver), clinical outcomes, or by a postoperative CBCT focusing on the membrane integrity in all 3D reconstruction planes. However, perforations occurring during or after a transcrestal approach could remain undetected with these techniques. Indeed, even post‐op CBCT scans exhibit an accuracy of only 60% with a predictive value of 64.3% [31]. In this context, endoscopy with a rhinolaryngoscope seems the most accurate way to assess the membrane integrity [11, 32]. However, a previous cadaver study using endoscopy after membrane elevation reported a perforation rate of up to 40% because all membrane damages, ranging from superficial wear to breaches leading to leakage between the maxillary sinus and oral cavity, were considered as perforations [11]. The Valsalva maneuver has limited reliability in frozen cadaver heads due to the absence of physiological pressure and compromised tissue elasticity after thawing. In the current study, the membrane integrity was assessed by post‐op CBCT only to mimic conventional clinical protocols, but the analyses were systematically performed in all the reconstruction planes.

Some studies about mechanical properties reported that the Schneiderian membrane could stretch up to 132%–241% of its original size, with a maximum elongation of 11 mm [19, 33]. The 3D elevations reported in the present study showed that beyond the volume obtained by the membrane stretching capabilities, a membrane detachment occurred upon the bone floor in each case and in all directions, despite variations in sinus morphology. The membrane detachment behavior was homogeneous in all sites in all anatomical subjects. However, the quantity of detachment in each direction varied from a site to another with the sinus morphology (wideness and architecture of the lateral wall). Interestingly, these 3D detachment values were proportional to the obtained elevation height. This study showed a predictable increase of 150% in detachment surface between the two groups (Figure 3).

The main perspective of this study is that a sinus augmentation volume (by 150% from 4 to 8 mm elevation height) could improve the bone reconstruction volume. Therefore, by increasing Schneiderian membrane elevation height up to 8 mm, the bone volume augmentation upon a residual bone height ≤ 5 mm would be similar to the volume obtained using a lateral approach. If confirmed by clinical studies, the indications of the transcrestal approach would no longer be limited to membrane elevation of 4 mm and to residual bone height of 7 and 9 mm [4, 20, 21].

In the early learning phase, inexperienced operators will often hesitate and use insufficient force (therefore no sinus floor fracture) or excessive or misdirected force (therefore membrane tearing). However, by following the strictly standardized protocol, an inexperienced practitioner may only need a few cases to master this technique. Experienced users adapt quickly to the new osteotomes, and their use is easier than that of regular osteotomes. No human clinical study directly analyzes or statistically correlates bone density with Schneiderian membrane elevation rates at the implant‐site level during transcrestal sinus lift procedures performed exclusively with osteotomes. However, transcrestal elevation on sites with denser bone than type IV should be facilitated with the new osteotomes.

Few comparative studies have statistically assessed membrane perforation rates across commercial osteotome systems, highlighting a need for further research. While innovations aim to reduce perforation, only limited data support the superiority of specific kits [11]. The use of a new‐generation osteotome with a hollow tip that disperses forces radially from the circular bone fragment appears to enhance sinus augmentation. This technique not only enables vertical membrane elongation—which, if excessive, may lead to overstretching and rupture—but also promotes a more uniform detachment of the membrane from the bone floor, potentially reducing the risk of perforation. Other minimally invasive techniques were described for sinus elevation. The piezo surgery is used to expose the maxillary sinus membrane from the alveolar ridge crest and simultaneously elevate the sinus floor by hydraulic pressure. However, these approaches are lacking large, high‐quality datasets for residual bone height of 4 mm [34]. The ballooning technique consist in screwing in a metal sleeve (2.6 mm in internal diameter) just 0.5 mm under the sinus floor. Subsequently, an inflatable balloon is inserted, extending 1–2 mm beyond the sleeve's tip. Balloon‐assisted techniques appear to have comparable perforation rates to osteotome lifts, though the data related to residual bone height of 4 mm are limited [35, 36, 37]. Osseodensification techniques using high‐speed densifying burs (Densah burs, Versah, Jackson, MI, USA) help preserve and compress bone in place. The presence of irrigation creates a hydraulic wave at the tip of the bur, which pushes the maxillary sinus membrane upwards. Technique‐matched data in this exact atrophic stratum are still sparse, but osseodensification burs appear to produce a perforation risk in the same range observed with osteotome lifts [38].

Frozen cadaver heads were used to safely and ethically evaluate the new osteotomes in a controlled, anatomically realistic environment, minimizing patient risk during early‐stage testing. This model allowed direct assessment of membrane integrity and instrument performance prior to clinical application. The main limitation of this study on cadaver is the possible change in tissue mechanical properties, as bone and Schneiderian membrane elasticity may differ from living tissues [11]. However, postmortem tissue changes in fresh and frozen specimens were shown to have little impact on the mechanical properties of tissue [39]. Moreover, some studies showed that freezing the Schneiderian membrane increased the tissue fragility and led to perforations when stretched [11, 19]. Therefore, the incidence of sinus perforation in previously frozen cadaver heads might be higher than in living humans [19]. To minimize the bias, only frozen fresh cadaver heads were included in the study. Another limitation of this study is the lack of comparison between this new protocol and membrane elevation using a regular osteotome. However, as the perforation rates with the hollowed osteotome were similar to the literature using a regular osteotome, the effectiveness of the technique is compatible with clinical use. Finally, endoscopy with a rhinolaryngoscope or a microscope assessment could have been a more accurate way to assess the membrane integrity [11, 32, 40]. Clinical studies are, however, needed to confirm the results of the present study.

## 5 Conclusion

With a transcrestal approach using this new‐generation osteotome, sinus membrane elevations of 8 mm were performed with a low perforation rate in cadavers after CBCT assessment. Further investigations should be conducted with endoscopy examination of membrane perforation and a control group using regular osteotomes. Moreover, clinical investigations will be needed to assess its safety, reproducibility, and predictability. However, this transcrestal elevation protocol could increase the indications of the technique, especially concerning single implant restorations and for frail patients with significant medical history for whom a lateral approach would be too invasive.

## Author Contributions

**Jean‐Christophe Coutant:** experiment, formal analysis, writing – original draft. **Adrien Naveau:** writing – review and editing. **Yves Lauverjat:** experiment, writing – original draft. **Bruno Ella:** conceptualization, methodology, writing – original draft.

## Ethics Statement

The authors have nothing to report.

## Consent

The authors have nothing to report.

## Conflicts of Interest

The authors declare no conflicts of interest.

## Supporting information


**Table S1:** Reporting of the CACTUS guidelines for the present study.

## Data Availability

The data that support the findings of this study are available on request from the corresponding author. The data are not publicly available due to privacy or ethical restrictions.
