# Supplementary material for: Schneiderian Membrane Elevation up to 8 mm by Transcrestal Technique Using Hollowed Osteotomes: A Human Cadaver Study
Source: Clin Implant Dent Relat Res. 2025 Oct 11;27(5):e70096. doi: 10.1111/cid.70096 (PMC12514859; doi:10.1111/cid.70096)
Supplement: Supplementary file 1 — Table S1: Reporting of the CACTUS guidelines for the present study. [file CID-27-0-s001.docx]

**Table 1.** Reporting of the CACTUS guidelines for the present study.

|  | **CACTUS Guidelines** | ***Report for the present study*** |
| --- | --- | --- |
| 1 | *Approval of the use of the bodies with a clear statement of the institute that approves their use.* | Approval of the Anatomy Laboratory (University of XXX) of the use of body donations, in accordance with the XXX Law. |
| 2 | *Number and gender of the bodies and/or organs used. If possible, report important clinical data such as BMI, basic medical history or previous non neglectable surgery.* | Thirty-four heads were used. Only the maxillary arches were used. |
| 3 | *State of conservation of corpses and/or parts of them, indicating the days of death before preservation.* | Heads were frozen 24 to 48h after death. |
| 4 | *In case of preparation and/or embalming of the body/organ, briefly indicate the methodology used (i.e. Fresh frozen, Thiel’s technique, etc.) and the time elapsed from the conservation procedure to the use of the corpse for training/study purposes.* | Heads were fresh frozen. |
| 5 | *Indicate the type of study for which they are used (anatomical study, surgical study, surgical training, device training, etc.).* | The samples were used for a surgical study in oral surgery (primary stability measurements of dental implants). |
| 6 | *The type of fluids other than water with which the bodies come into contact during the study (i.e. Saline solution 0.9%, formaldehyde, etc.) might be a useful additional but not mandatory information.* | Irrigation with saline solution 0,9% was performed during drilling. |
| 7 | *If cadaver specimens are sampled for pathological evaluation, the type and method of sampling might be a useful additional but not mandatory information.* | Not applicable.  Specimens were not sampled for pathological evaluation. |
| 8 | *Indicate the number and qualification of investigators/trainees actively involved in the cadaver study/training. Only people involved in organization and training should be present in the activities, since the use of human bodies for study should be treated with all the ethics they deserve.* | All investigators are qualified surgeons in Periodontology and/or Oral Surgery, being either associate professors or full professor working at both university and hospital. |
| 9 | *Provide brief outcomes in terms of satisfaction in the use of the cadaver model through a short questionnaire to be administered to the trainees/investigators and comparing the different models used in the study (i.e. Thiel fixed cadavers vs fresh frozen; i.e. human cadaver model used vs another biological or non-biological model used). If the human cadaver is the only model used in the study provide an overall comment on satisfaction compared to that expected. When it is possible, also report objective data on the usefulness of the training model.* | The human cadaver was the only model used in the study. |
